# Supplementary material for: In Vitro Antibacterial Activity of Biological-Derived Silver Nanoparticles: Preliminary Data
Source: Vet Sci. 2020 Jan 23;7(1):12. doi: 10.3390/vetsci7010012 (PMC7157719; doi:10.3390/vetsci7010012)
Supplement: Supplementary file 1 [file vetsci-07-00012-s001.pdf]

veterinary sciences

Article

In vitro antibacterial activity of biological-derived silver nanoparticles: preliminary data

Gabriele Meroni [1,2,*], Joel F. Soares Filipe[1] and Piera A. Martino [1]

1 Department of Veterinary Medicine, Università degli Studi di Milano, Lodi, 26900, Italy
2 Department of Biomedical Sciences for Health, Università degli Studi di Milano, Milano, 20133, Italy
* Correspondence: gabriele.meroni@unimi.it; Tel.: +39 0250315326

Received: date; Accepted: date; Published: date

Supplementary materials

Biofilm forming ability of *Pseudomonas* and *Staphylococcus* strains

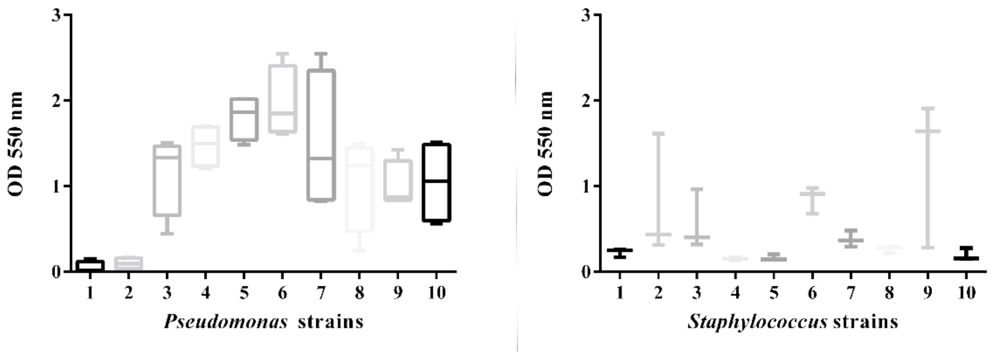

Figure S1. Optical densities of all the strains as measured following the method described by Stepanović. 2007. After MtP assay, among the 10 *Pseudomonas* strains, 8 were considered strongly biofilm producers. Among the 10 *S. pseudintermedius* strains 4 resulted non biofilm-producers, 2 weakly producers and 4 strongly producers.

© 2020 by the authors. Licensee MDPI, Basel, Switzerland. This article is an open access article distributed under the terms and conditions of the Creative Commons Attribution (CC BY) license (http://creativecommons.org/licenses/by/4.0/).
